# Glomus Tumor of the Stomach: GI Image

**DOI:** 10.1007/s11605-016-3321-x

**Published:** 2016-11-14

**Authors:** Carolina Castro Ruiz, Gabriele Carlinfante, Maurizio Zizzo, Alessandro Giunta, Roberto Ronzoni, Francesco Azzolini, Claudio Pedrazzoli

**Affiliations:** 1 Department of General Surgery, C.S. Surgical Oncology and Reconstructive Surgery, Azienda Ospedaliera—IRCCS Arcispedale Santa Maria Nuova, Viale Umberto I, 50 -42123 Reggio Emilia, Italy; 2 Pathology Unit, Azienda Ospedaliera—IRCCS Arcispedale Santa Maria Nuova, Reggio Emilia, Italy; 3 Unit of Gastroenterology and Digestive Endoscopy, Azienda Ospedaliera—IRCCS Arcispedale Santa Maria Nuova, Reggio Emilia, Italy

**Keywords:** Glomus tumor, Endoscopic ultrasound-fine needle aspiration, Stomach, Gastric, Cytology, Laparoscopic surgery

## Clinical Data

**Fig. 1:**
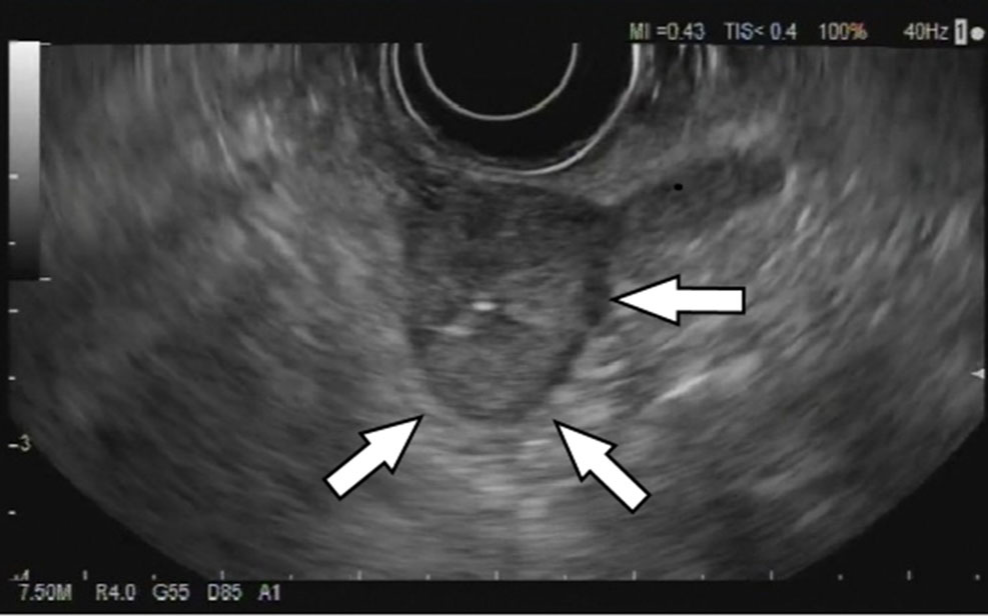
Endoscopic ultrasound. Presence of a lesion rising from the IV layer of the stomach wall (*white arrows*)

**Fig. 2:**
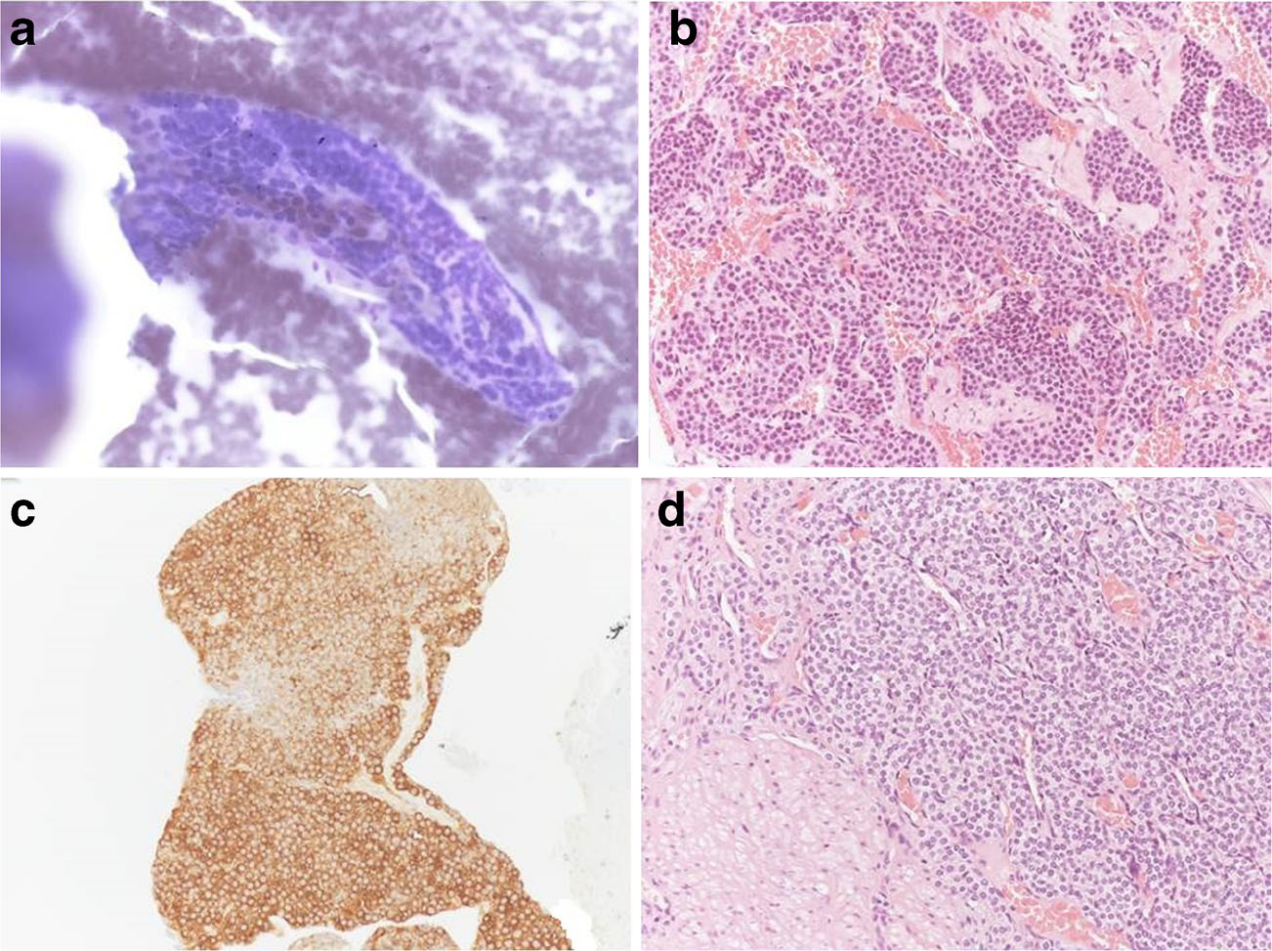
Cytological and histological preparations. **a** Cytological smear showed a solid cluster of tumor cells with round nuclei and scant cytoplasm. Giemsa stain, 200× magnification. **b** Cell-block material showed sheets of rounded cells with small nuclei and sharply defined cell borders. Hematoxylin Eosin stain, 200× magnification. **c** Immunochemistry for smooth muscle actin was strongly and diffusely positive. 200× magnification. **d** Histological section of gastric tumor showed cellular nodules with prominent slit-like blood vessels adjacent to bands of smooth muscle of muscolaris propria

**Fig. 3:**
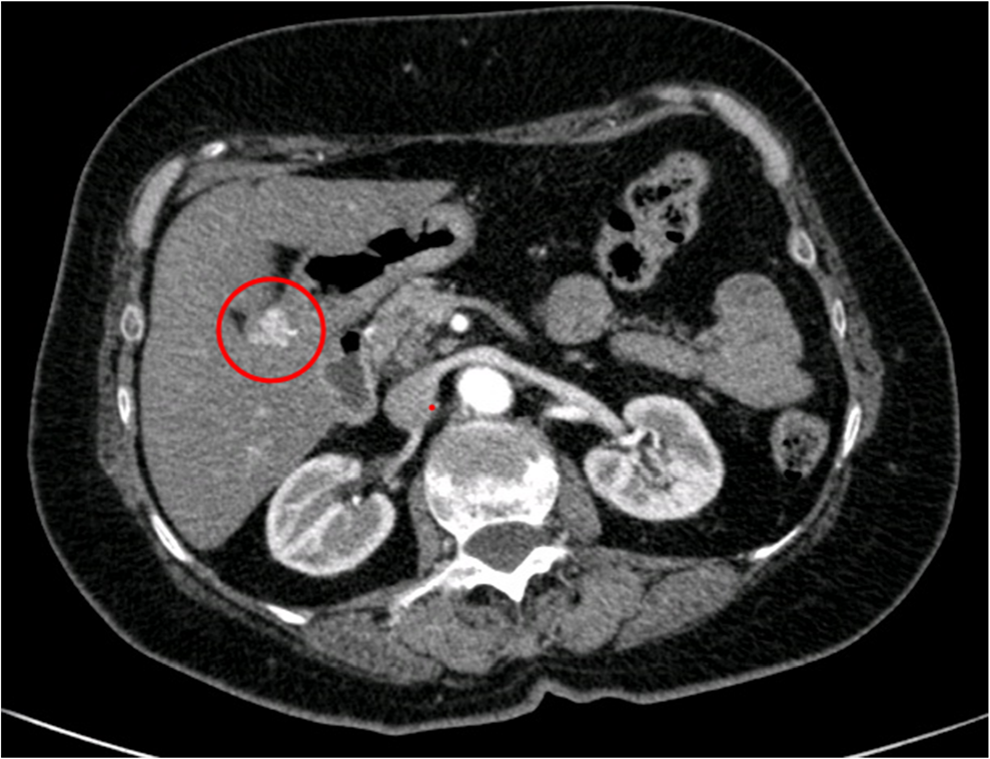
Computed tomography. Enhancement of the lesion in arterial phase that shows the lesion within the gastric wall

**Fig. 4:**
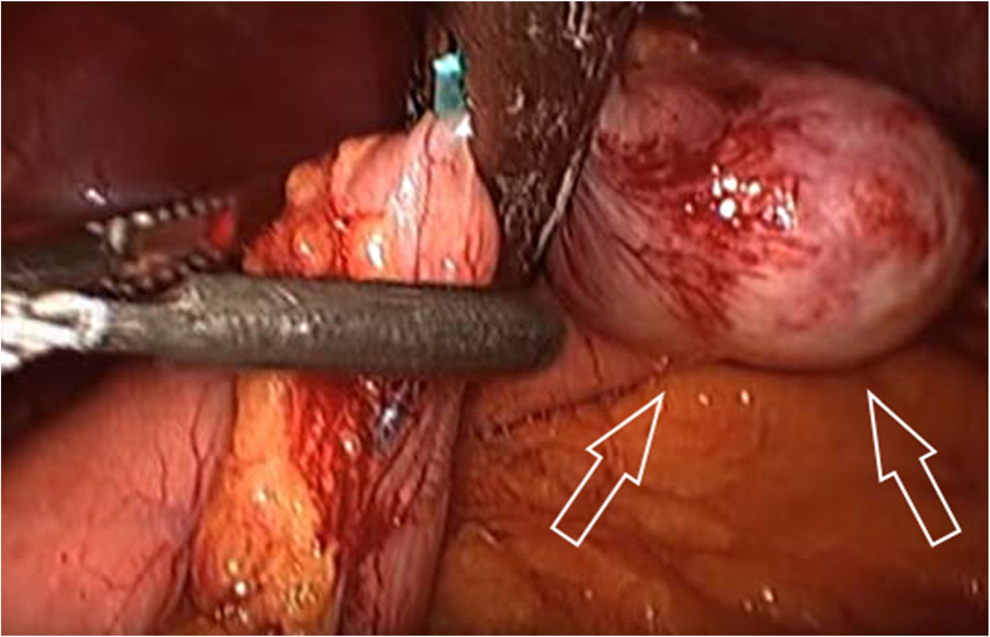
Glomus tumor (*white arrows*) being resected by an endoscopic stappler

A 70-year-old female presented to our attention with an incidental finding, of a gastric mass, during videolaparoscopic cholecystectomy; the surgeon described a mass forming lesion within the gastric wall that did not erose the serosa. The patient underwent different gastroscopies, and during the last gastroscopic control, the endoscopist found a significant increasement of the well-known mass (2 cm in diameter), located in the gastric antrum nearby the pylorum laying in the greater curvature. According to the macroscopic findings, our first diagnostic hypothesis was of GIST. Then, an EUS-FNA (Fig. 1) was performed with on-site cytopathology assistance to evaluate the adequacy of material. The cytopathology smear showed a population of uniform, round epithelioid cells, with relatively small nucleoli and variable eosinophilic cytoplasm, which stained for smooth muscle actin but were negative for desmin, chromogranin, synapthophisin, and keratin (Fig. 2). A final cytological diagnosis of glomus tumor (GT) of the stomach was obtained. The patient underwent a CT scan (Fig. 3) that confirmed the presence of a hyperdense lesion of about 14 mm, in the absence of lymphadenomegaly or metastatic disease. There weren’t any contraindications for surgery, so we decided to perform a gastric laparoscopic wedge resection of the lesion (Fig. 4). Laparoscopy is a good minimally invasive technique in case of small and benign tumors, which allowed us to discharge the patient in the fourth postoperative day. Macroscopically, the lesion measured 2.5 × 1.6 × 1.2 cm with well-defined borders and rubbery consistency. The histological examination confirmed the cytological diagnosis of GT of the stomach with surgical margins free of disease.

## Discussion

The glomus apparatus consists of three vascular components: an afferent artery separated from an efferent venule by convoluted channels. They are commonly observed wherever arteriovenous anastomoses functioning without an intermediary capillary bed are present, are sensitive to temperature variation, and play a role in regulating arterial blood flow. Glomus tumors (GTs) are commonly described in the extremities and are less common in the visceral organs.^1^ GT of the stomach are usually bigger than the cutaneous forms measuring on average 2–2.5 cm in size and are more commonly located in the antrum. Clinical presentation may vary, going from a recovery in the emergency room due to gastrointestinal bleeding (hematemesis and/or melena) to an epigastric discomfort or chronic anemia. At endoscopy, glomus gastric tumors look as submucosal masses with a smooth surface or ulceration on the top often misdiagnosed as gastric cancer or gastric stromal tumor. EUS-FNA is an effective method to obtain cytological material of gastric submucosal neoplasms with an accuracy rate of 95.6% and represents a rapid cost-effective method to differentiate GTs from more aggressive gastric tumors.^2^

The main diagnostic modalities for evaluating submucosal tumors are endoscopic ultrasound (EUS) and CT scan (computed tomography) but is the pathological exam that can really give us an accurate diagnosis of GT, specially, in those cases in which diagnosis through imaging is not feasible. GTs have shown positivity in the histoimmunochemical panel of tissue markers such as smooth muscle actin and type IV collagen. Other markers, including desmin, cytokeratin (AE1/AE3b), S-100 protein, creatine kinase, C-KIT (CD-117), CD34, DOG1 protein (K9), chromogranin A, p53 protein, and neuron specific enolase, were negative. Immunohistochemical markers are key to diagnose these tumors in order to differentiate GTs from stromal, vascular, and lymphatic tumors.^3^

GTs are essentially benign in nature; therefore, wedge resection or subtotal gastrectomy to remove the tumor and its envelope can cure the patient and supply a good prognosis.

## Conclusions

At the moment, there are no clear guidelines regarding the optimal therapy for glomus tumors of the stomach. We believe that the gold standard during diagnosis ought to be EUS-FNA which gives us a more accurate diagnosis of the lesion. It is our belief that after diagnosis has been made, considering the benign behavior of this lesion and in the absence of clear malignancy parameters, complete surgical removal of the tumor, preferably by laparoscopic (wedge resection) technique, is a reasonable therapeutical choice.

## Abbreviations

EUS-FNA: Endoscopic ultrasound-fine needle aspiration
CT: Computed tomography
GIST: Gastrointestinal stromal tumors
MRI: Magnetic resonance imaging
